# Charting the COVID-19 vaccination journey in Saudi Arabia: Insights into post-vaccination adverse effects and immunization dynamics

**DOI:** 10.3389/fphar.2025.1561410

**Published:** 2025-04-28

**Authors:** Gehad M. Subaiea, Nawaf Alkhateeb, Faisal Sahman, Abdulrahman Alsudayri, Abdulkarim M. Almudayni, Hamoud Alrashidi, Abdulrahman M Alshammari, Abdulwahab Alamri, Sultan Almuntashiri, Arshad Hussain, Heba Ali Khloofi, Sirajudheen Anwar

**Affiliations:** ^1^ Department of Pharmacology and Toxicology, College of Pharmacy, University of Hail, Hail, Saudi Arabia; ^2^ College of Pharmacy, University of Hail, Hail, Saudi Arabia; ^3^ Department of Clinical Pharmacy, College of Pharmacy, University of Hail, Hail, Saudi Arabia

**Keywords:** formatted: numbering: continuous COVID-19 vaccination, Saudi Arabia, cross-sectional study, socio demographic characteristics, adverse effects, vaccine administration, infection rates

## Abstract

**Background:**

The current study evaluated the effects of different COVID-19 vaccines on Saudi Arabian residents, focusing on their safety, acceptance, and effectiveness. Gaining a better knowledge of these vaccination results will help develop more successful public health initiatives and increase confidence in vaccination campaigns throughout the Kingdom.

**Methods:**

A cross-sectional study was conducted with 401 participants from diverse backgrounds, covering different ages, genders, nationalities, weights, and education levels. The survey gathered information about participants’ health conditions, their vaccines, side effects, and infection rates before and after vaccination. The data were analyzed to compare vaccine preferences, side effects, and infection trends overtime.

**Results:**

Sociodemographic-wise, most participants were men (62.84%) and Saudi nationals (96.01%), showing significant differences by gender and nationality (P < 0.001). The largest age group was 21–30 years (45.89%, P < 0.001), with 66.58% being university graduates (P < 0.001). Pfizer/BioNTech was the top choice across all doses, with 83.46% receiving it for the first dose, 78.1% for the second, and 39.28% for the third, reflecting a clear preference over other vaccines (P < 0.001). Pfizer/BioNTech recipients reported side effects after the first dose in 36.53% of cases, but only 1.86% needed medical help. Vaccination significantly reduced infection rates: Pfizer/BioNTech dropped infection rates from 43.18% to 8.33% after the third dose (P < 0.001), while Oxford/AstraZeneca saw rates fall from 12.88% to 0.76% after the third dose, but did not reach significance (P = 0.34). Overall, vaccinated individuals had much lower infection rates (28.17%) than among unvaccinated ones (100%), with a P-value of 0.020.

**Conclusion:**

Our results concluded that Saudi Arabia’s vaccination campaign has proven effective, especially after the second and third doses. Pfizer/BioNTech was the most preferred vaccine, demonstrating strong efficacy and safety, which helped build public confidence. Ongoing monitoring is crucial to maintaining pandemic control, post-marketing and public health strategies.

## Background

The ongoing coronavirus disease 2019 (COVID-19) pandemic, caused by the SARS-CoV-2 virus, continues to present a significant global health challenge.

As of 19 September 2023, the World Health Organization (WHO) recorded over 770 million confirmed cases and about 6.9 million deaths linked to COVID-19 (Valderrama-Beltran et al., 2023).

This pandemic has not only resulted in a large number of illnesses and fatalities, but it has also caused major socioeconomic disruptions worldwide, disrupting healthcare systems, economies, and everyday life (Ulloque-Badaracco et al., 2024). The persistent burden of COVID-19 is demonstrated by the continuous updates from health organizations, which highlight the need for sustained public health measures and efforts to mitigate the spread of the virus and its variants (de-Oliveira-Pinto et al., 2022). Furthermore, the impact of COVID-19 extends beyond immediate health concerns, as it has been associated with long-term health complications and has exacerbated existing health disparities across different populations (Yan et al., 2022). The WHO’s data serves as a crucial reminder of the pandemic’s scale and the importance of ongoing vigilance and response strategies to manage its effects effectively (Kim, 2024).

Initially identified in Wuhan, China, in late 2019, this novel virus rapidly spread worldwide, resulting in the WHO declaring it a pandemic by March 2020 (Cucinotta and Vanelli, 2020). In the early phase of the outbreak, preventive measures such as lockdowns, social distancing, and travel restrictions were the primary responses to curbing viral transmission, given the lack of effective treatments or vaccines at the time (Murphy et al., 2023). These measures, however, led to socio-economic disruptions, emphasizing the urgent need for a more sustainable solution—vaccination.

Vaccination quickly emerged as the most promising intervention to control the spread of COVID-19 and reduce associated morbidity and mortality (Ali et al., 2023). By early 2021, several vaccine candidates had successfully passed clinical trials and received emergency use authorization (EUA). The Pfizer/BioNTech vaccine, approved for emergency use in December 2020 by the U.S. Food and Drug Administration (FDA), demonstrated a 95% efficacy rate, while Moderna’s vaccine followed with an efficacy rate of 94.1% (Baden et al., 2021) The Pfizer/BioNTech vaccine requires two doses administered at least 21 days apart, while the Moderna vaccine necessitates a similar two-dose regimen with a 28-day interval (Moghadas et al., 2021).

In Saudi Arabia, the Ministry of Health initiated a nationwide vaccination campaign that prioritized healthcare professionals, especially emergency responders in vital areas, individuals aged 60 and above, obese, organ transplant recipients who are taking immunosuppressive drugs, and those with chronic health conditions, reflecting a targeted approach to achieve high vaccination coverage (Noushad et al., 2021) As of early 2023, approximately 70% of the Saudi population had received at least one dose of a COVID-19 vaccine, with booster doses administered to over 30% of the population (Alshahrani et al., 2023).

Despite these advancements, challenges such as vaccine hesitancy persisted, influenced by factors like public perceptions, misinformation, and varying levels of vaccine (Bond et al., 1998). Understanding vaccine hesitancy is crucial, as it represents a significant barrier to achieving optimal vaccine coverage and herd immunity (Gust et al., 2003). Defined as a delay in acceptance or outright refusal of available vaccines, vaccine hesitancy is influenced by individual perceptions of the vaccine’s safety, efficacy, and potential adverse effects (MacDonald, 2015).

Studies have identified demographic factors like age, gender, and educational level as influential in shaping vaccine acceptance in Saudi Arabia. Younger individuals and those with lower educational attainment have shown greater hesitancy compared to other (Almalki et al., 2023). Additionally, adverse events following immunization (AEFI) have been cited as one of the leading causes of hesitancy (Fadhel, 2021). The WHO defines AEFI as “any untoward medical occurrence which follows immunization and which does not necessarily have a causal relationship with the usage of the vaccine” (Alhazmi et al., 2021). These events can range from mild side effects, such as soreness at the injection site or fever, to more severe but rare reactions, including allergic responses (Alhazmi et al., 2021).

This study sought to assess the adverse effects (AEs) linked to various COVID-19 vaccines and dosages among vaccinated individuals in Saudi Arabia. The study analyzed factors affecting vaccine hesitancy, emphasizing demographic characteristics such as age, gender, and pre-existing health conditions. This study aimed to assess the effectiveness of vaccination in lowering reinfection rates during the second and third waves of COVID-19 in Saudi Arabia, while also examining its role in preventing hospitalizations and severe outcomes in individuals with comorbidities. The current research will enhance the understanding of COVID-19 vaccine safety, public acceptance, and the effectiveness of vaccination campaigns within the context of Saudi Arabia. Clear and reliable information can enhance public trust in vaccine safety, subsequently increasing vaccination rates.

## Methods

### Data and data processing

This research was conducted as a cross-sectionalstudy. Location and Duration: The study was conducted in various locations across the Hail region, including residential areas, workplaces, malls, and public health facilities between April 2022 – October 2022. Sample Size Calculation: Using the Raosoft sample size calculator [http://www.raosoft.com/samplesize.html], a minimum sample size of 377 were determined. This calculation was based on a target population of 20,000 individuals aged 16 to 74, with a 95% confidence level, a 5% margin of error, and an assumed response distribution of 50%.

$$n=Z^{2}\;P\left(1-P\right)/d2$$

Where, n = required sample size, P = disease prevalence Z = confidence level, and d = margin of error. Additionally, using a cluster sampling methodology, a sampling error of 6% was computed, hence the total sample size was 401.

The primary instrument utilized in this research was a questionnaire that was administered in both Arabic and English. The questionnaire was developed based on a comprehensive review of the literature and refined through expert consultation. For face validity, a panel of five public health and vaccination specialists evaluated the draft to ensure that each item was clear and relevant. For content validity, a literature review was conducted to verify that all critical aspects, including vaccination outcomes, potential adverse effects, and sociodemographic variables, were adequately addressed. The validation process employed Cronbach’s alpha analysis and Bartlett’s test of sphericity, and involved an initial sample of 40 responses, yielding a Cronbach’s alpha value of 0.87 and a significant Bartlett’s test of sphericity (p < 0.001).

The study included both male and female participants aged 16 years and above from the general public who were willing to participate voluntarily. The participants were selected from the Hail region using a cluster random sampling method at locations such as malls, public health centers, and banks. Data was gathered electronically using Google Forms with 15 or more questions. To ensure confidentiality, participation was entirely voluntary, with identities kept anonymous. The survey link was shared via digital platforms like free cross-platform messaging apps, workgroups, residential networks, and public spaces. Additional data was collected from public health centers. The purpose of the study was clearly communicated to participants, and completing the survey implied consent. Responses were electronically transferred to Google Sheets for analysis.

Data collection for this study was carried out through an online survey, using a structured questionnaire designed in Google Forms. The survey aimed to assess COVID-19 reinfection rates, vaccine side effects, and the overall impact of vaccination on participants’ health. To ensure wide participation, the survey link was distributed digitally through various platforms like messaging apps, emails, and community groups. The survey was shared in public spaces, such as malls, banks, workplaces, residential communities, and public health centers across the Hail region. By using a digital approach, we were able to reach a broader audience quickly while maintaining participants’ anonymity. The purpose of the study was clearly communicated to participants, and their willingness to complete the survey was taken as implied consent. The questionnaire was developed based on the COVID Symptom Study, drawing from well-established research to ensure thorough data collection. It started with basic demographic questions, asking about the participants’ age, gender, place of residence, and ethnic background. Participants were also asked to provide information on their health, including self-reported height, weight (for BMI calculations), smoking status, and any existing health conditions. Key comorbidities explored included diabetes, cancer, eczema, cardiovascular disease, respiratory illnesses, kidney disease, and allergies. Employment details were also captured, with special attention given to healthcare workers to understand how vaccination affected this high-risk group. Participants were then asked to share details about their vaccination status. Questions covered the type of vaccine received—Pfizer/BioNTech, Oxford/AstraZeneca, Moderna, Janssen (Johnson & Johnson), or other available vaccines—and whether they had received the first, second, or booster dose. This information helped us compare side effects across different vaccine types and doses. To gather detailed information on side effects, the survey asked about any symptoms experienced within the first 8 days following vaccination. Participants could report systemic side effects such as headache, fatigue, chills, fever, diarrhea, joint pain, muscle pain, or nausea. Local side effects, like pain, swelling, tenderness, redness, itching, warmth, or swollen armpit glands at the injection site, were also included. For those who experienced no symptoms, the option to leave the checkboxes empty was provided. This design ensured that all possible reactions were captured, offering a clear picture of the frequency and intensity of vaccine side effects.

### Statistical analysis

Responses were automatically recorded in Google Sheets, making data processing straightforward. To ensure accuracy, the analysis was performed using the online tool available at https://www.socscistatistics.com/tests/, and the results were cross-verified using established statistical software like SPSS and Minitab. Descriptive statistics were used to summarize the data, while the Chi-square test of independence helped explore relationships between different variables, such as vaccine type, side effects, and demographic factors, with a significance level set at 0.05. The survey also included questions about post-vaccination symptoms that could suggest potential reinfection, such as fever, cough, loss of taste or smell, and shortness of breath. Participants were encouraged to describe the timing and severity of these symptoms to better understand the vaccine’s role in reducing reinfection rates. Additionally, a section dedicated to quality of life explored how vaccination affected daily activities, work routines, and overall wellbeing. This approach aimed to capture not only the immediate side effects of vaccination but also its broader impact on participants’ lifestyles and health.

## Results

As summarized in Table 1, which represents the general sociodemographic characteristics of the study participants, most of the study participants were male (62.84%), while females made up (37.16%) of the group. Nearly all participants (96.01%) were Saudi nationals, with a small fraction (3.99%) being non-Saudis. In terms of age, the majority were young adults aged 21–30 years (45.89%), followed by those in the 31–40 age group (18.45%). The most common weight range was 51–75 kg (45.39%), with another significant portion in the 76–100 kg range (37.66%). When it comes to education, a large portion of participants had university degrees (66.58%), while about a fifth had finished high school (21.20%). A considerable number were students (36.66%), while others were employed in government (27.43%) or private sectors (18.45%). Most participants reported a monthly income of less than 5,000 SAR (53.37%), while 19.95% had incomes between 10,000 and 15,000 SAR. About 12.47% earned between 5,000 and 10,000 SAR, and 10.47% fell in the 15,000 to 20,000 SAR range. Only a small percentage of participants earned 20,000. The wide variety of socio-demographic factors, such as age groups, educational attainment, work statuses, and income ranges, demonstrates the extensive diversity among the Saudi population.

**TABLE 1:** Demographic characteristics of the study participants.

Demographic data					
Socio-demographic characteristics	N	(N, %)	%	95% CI	Sig
Gender					
Male	252	(252, 62.84%)	62.84%	0.58–0.67	P < 0.001
Female	149	(149, 37.16%)	37.16%	0.32–0.42	
Nationality					
Saudi	385	(385, 96.01%)	96.01%	0.94–0.97	P < 0.001
Non-Saudi	16	(16, 3.99%)	3.99%	0.03–0.07	
Age Group (Years)					
14–20	61	(61, 15.21%)	15.21%	0.12–0.19	P < 0.001
21–30	184	(184, 45.89%)	45.89%	0.41–0.50	
31–40	74	(74, 18.45%)	18.45%	0.15–0.23	
41–50	54	(54, 13.47%)	13.47%	0.10–0.17	
51–60	25	(25, 6.23%)	6.23%	0.04–0.09	
61–100	3	(3, 0.75%)	0.75%	0.002–0.02	
Weight (kg)					
34–50	42	(42, 10.47%)	10.47%	0.08–0.14	P < 0.001
51–75	182	(182, 45.39%)	45.39%	0.41–0.50	
76–100	151	(151, 37.66%)	37.66%	0.33–0.42	
101–125	18	(18, 4.49%)	4.49%	0.03–0.07	
126–150	6	(6, 1.5%)	1.50%	0.007–0.03	
151–175	2	(2, 0.5%)	0.50%	0.0001–0.01	
Education level					
Diploma	5	(5, 1.25%)	1.25%	0.005–0.028	P < 0.001
High School	85	(85, 21.2%)	21.20%	0.17–0.25	
Intermediate	8	(8, 2%)	2.00%	0.01–0.03	
University graduate	267	(267, 66.58%)	66.58%	0.62–0.71	
PG (Masters and/or PhD)	32	(32, 7.98%)	7.98%	0.57–0.11	
I can read (Literate)	4	(4, 1%)	1.00%	0.003–0.025	
Employment Status					
Government	110	(110, 27.43%)	27.43%	0.23–0.32	P < 0.001
Private	74	(74, 18.45%)	18.45%	0.15–0.23	
Retired	12	(12, 2.99%)	2.99%	0.017–0.051	
Student	147	(147, 36.66%)	36.66%	0.32–0.41	
Housewife	22	(22, 5.49%)	5.49%	0.04–0.08	
Unemployed	35	(35, 8.73%)	8.73%	0.06–0.11	
Others	1	(1, 0.25%)	0.25%	0.000–0.01	
Avg. monthly Income (SAR)					
*<*5,000	214	(214, 53.37%)	53.37%	0.48–0.58	P < 0.001
5,000 to *<*10,000	50	(50, 12.47%)	12.47%	0.09–0.16	
10,000 to *<*15,000	80	(80, 19.95%)	19.95%	0.16–0.24	
15,000 to *<*20,000	42	(42, 10.47%)	10.47%	0.08 0.14	
20,000 & Above	15	(15, 3.74%)	3.74%	1.2–0.06	

In terms of health conditions, most participants were free from chronic diseases, with 86.11% of men and 79.19% of women reporting no health issues. However, among those who did have chronic conditions, diabetes was the most common, affecting 10.74% of women and 6.75% of men, Table 2. Hypertension was more frequent in women (8.05%) than in men (2.38%), while heart disease was also higher among women (3.36%) compared to men (0.4%). Saudi nationals showed higher rates of chronic conditions, particularly diabetes (8.57%) and hypertension (5.45%). As expected, the prevalence of chronic diseases increased with age; for example, nearly half (48%) of participants aged 51–60 had diabetes, while 36% had hypertension. Similarly, those with higher body weights had a greater tendency towards chronic diseases, with 22.22% of participants in the 101–125 kg range being overweight or obese. Educational levels also played a role, as individuals with lower education reported higher rates of diabetes (12.5% among those with intermediate education). Retired participants experienced the highest rates of both diabetes (66.67%) and hypertension (66.67%), while chronic disease rates were lower among participants with lower incomes.

**TABLE 2:** Chronic disease prevalence and sociodemographic factors among participants.

Socio-demographic characteristics	Heart diseases	Diabetes mellitus	Hypertension	Kidney diseases	Overweight-obesity	Liver diseases	HIV	No chronic disease	Others
Gender									
Male	1 (0.4%)	17 (6.75%)	6 (2.38%)	1 (0.4%)	7 (2.78%)	1 (0.4%)	1 (0.4%)	217 (86.11%)	4 (1.59%)
Female	5 (3.36%)	16 (10.74%)	12 (8.05%)	1 (0.67%)	2 (1.34%)	0 (0%)	0 (0%)	118 (79.19%)	6 (4.03%)
P-Value	P < 0.001	P < 0.001	P < 0.001	P < 0.001	P < 0.001	P < 0.001	P < 0.001	P < 0.001	P < 0.001
Nationality									
Saudi	6 (1.56%)	33 (8.57%)	21 (5.45%)	2 (0.52%)	8 (2.08%)	1 (0.26%)	1 (0.26%)	320 (83.12%)	10 (2.6%)
Non-Saudi	0 (0%)	0 (0%)	0 (0%)	0 (0%)	1 (6.25%)	0 (0%)	0 (0%)	15 (93.75%)	0 (0%)
P-Value	P < 0.001	P < 0.001	P < 0.001	P < 0.001	P < 0.001	P < 0.001	P < 0.001	P < 0.001	P < 0.001
Age Group (Years)									
14–20	0 (0%)	2 (3.28%)	1 (1.64%)	0 (0%)	1 (1.64%)	0 (0%)	0 (0%)	56 (91.8%)	1 (1.64%)
21–30	1 (0.54%)	5 (2.72%)	0 (0%)	1 (0.54%)	5 (2.72%)	1 (0.54%)	0 (0%)	166 (90.22%)	6 (3.26%)
31–40	3 (4.05%)	4 (5.41%)	2 (2.7%)	0 (0%)	1 (1.35%)	0 (0%)	0 (0%)	65 (87.84%)	2 (2.7%)
41–50	1 (1.85%)	7 (12.96%)	6 (11.11%)	0 (0%)	1 (1.85%)	0 (0%)	1 (1.85%)	39 (72.22%)	1 (1.85%)
51–60	1 (4%)	12 (48%)	9 (36%)	0 (0%)	1 (4%)	0 (0%)	0 (0%)	9 (36%)	0 (0%)
61–100	0 (0%)	3 (100%)	3 (100%)	1 (33.33%)	0 (0%)	0 (0%)	0 (0%)	0 (0%)	0 (0%)
P-Value	P < 0.001	P < 0.001	P < 0.001	P < 0.001	P < 0.001	P < 0.001	P < 0.001	P < 0.001	P < 0.001
Weight (kg)									
34–50	0 (0%)	1 (2.38%)	0 (0%)	0 (0%)	0 (0%)	0 (0%)	0 (0%)	39 (92.86%)	2 (4.76%)
51–75	5 (2.75%)	11 (6.04%)	10 (5.49%)	1 (0.55%)	0 (0%)	1 (0.55%)	1 (0.55%)	156 (85.71%)	5 (2.75%)
76–100	0 (0%)	19 (12.58%)	11 (7.28%)	1 (0.66%)	2 (1.32%)	0 (0%)	0 (0%)	124 (82.12%)	3 (1.99%)
101–125	1 (5.56%)	2 (11.11%)	0 (0%)	0 (0%)	4 (22.22%)	0 (0%)	0 (0%)	11 (61.11%)	0 (0%)
126–150	0 (0%)	0 (0%)	0 (0%)	0 (0%)	3 (50%)	0 (0%)	0 (0%)	3 (50%)	0 (0%)
151–175	0 (0%)	0 (0%)	0 (0%)	0 (0%)	0 (0%)	0 (0%)	0 (0%)	2 (100%)	0 (0%)
P-Value	P < 0.001	P < 0.001	P < 0.001	P < 0.001	P < 0.001	P < 0.001	P < 0.001	P < 0.001	P < 0.001
Education level									
Diploma	0 (0%)	0 (0%)	0 (0%)	0 (0%)	0 (0%)	0 (0%)	0 (0%)	5 (100%)	0 (0%)
High School	1 (1.18%)	7 (8.24%)	8 (9.41%)	1 (1.18%)	0 (0%)	0 (0%)	0 (0%)	69 (81.18%)	2 (2.35%)
Intermediate	0 (0%)	0 (0%)	1 (12.5%)	0 (0%)	0 (0%)	0 (0%)	0 (0%)	7 (87.5%)	0 (0%)
University graduate	5 (1.87%)	20 (7.49%)	8 (3%)	0 (0%)	8 (3%)	1 (0.37%)	1 (0.37%)	225 (84.27%)	8 (3%)
PG (Masters and/or PhD)	0 (0%)	3 (9.38%)	1 (3.13%)	0 (0%)	1 (3.13%)	0 (0%)	0 (0%)	28 (87.5%)	0 (0%)
I can read (Literate)	0 (0%)	3 (75%)	3 (75%)	1 (25%)	0 (0%)	0 (0%)	0 (0%)	1 (25%)	0 (0%)
P-Value	P < 0.001	P < 0.001	P < 0.001	P < 0.001	P < 0.001	P < 0.001	P < 0.001	P < 0.001	P < 0.001
Employment Status									
Government	3 (2.73%)	12 (10.91%)	6 (5.45%)	0 (0%)	2 (1.82%)	0 (0%)	1 (0.91%)	88 (80%)	1 (0.91%)
Private	1 (1.35%)	4 (5.41%)	0 (0%)	1 (1.35%)	0 (0%)	0 (0%)	0 (0%)	68 (91.89%)	1 (1.35%)
Retired	0 (0%)	8 (66.67%)	8 (66.67%)	0 (0%)	1 (8.33%)	0 (0%)	0 (0%)	3 (25%)	0 (0%)
Student	1 (0.68%)	5 (3.4%)	1 (0.68%)	0 (0%)	5 (3.4%)	1 (0.68%)	0 (0%)	131 (89.12%)	4 (2.72%)
Housewife	1 (4.55%)	2 (9.09%)	5 (22.73%)	1 (4.55%)	0 (0%)	0 (0%)	0 (0%)	15 (68.18%)	1 (4.55%)
Unemployed	0 (0%)	2 (5.71%)	1 (2.86%)	0 (0%)	1 (2.86%)	0 (0%)	0 (0%)	29 (82.86%)	3 (8.57%)
Others	0 (0%)	0 (0%)	0 (0%)	0 (0%)	0 (0%)	0 (0%)	0 (0%)	1 (100%)	0 (0%)
P-Value	P < 0.001	P < 0.001	P < 0.001	P < 0.001	P < 0.001	P < 0.001	P < 0.001	P < 0.001	P < 0.001
Avg. monthly Income (SAR)									
*<*5,000	2 (0.93%)	9 (4.21%)	7 (3.27%)	1 (0.47%)	5 (2.34%)	1 (0.47%)	0 (0%)	185 (86.45%)	10 (4.67%)
5,000 to *<*10,000	0 (0%)	7 (14%)	5 (10%)	1 (2%)	1 (2%)	0 (0%)	0 (0%)	39 (78%)	0 (0%)
10,000 to *<*15,000	2 (2.5%)	8 (10%)	5 (6.25%)	0 (0%)	2 (2.5%)	0 (0%)	1 (1.25%)	66 (82.5%)	0 (0%)
15,000 to *<*20,000	2 (4.76%)	6 (14.29%)	2 (4.76%)	0 (0%)	1 (2.38%)	0 (0%)	0 (0%)	33 (78.57%)	0 (0%)
20,000 & Above	0 (0%)	3 (20%)	2 (13.33%)	0 (0%)	0 (0%)	0 (0%)	0 (0%)	12 (80%)	0 (0%)
P-Value	P < 0.001	P < 0.001	P < 0.001	P < 0.001	P < 0.001	P < 0.001	P < 0.001	P < 0.001	P < 0.001

The prevalence of chronic diseases among study participants showed variations across sociodemographic factors. Males generally reported fewer chronic conditions than females, with a significant difference across diseases such as diabetes, hypertension, and liver diseases (P < 0.001). Saudis had a higher prevalence of heart diseases, diabetes, and hypertension compared to non-Saudis, though most non-Saudis reported no chronic diseases (93.75%, P < 0.001). Younger participants (aged 14–30) had lower rates of chronic diseases, while those aged 51 and above reported higher rates of diabetes and hypertension (P < 0.001). Regarding weight, participants within the 76–100 kg range showed higher rates of diabetes and hypertension, while the 126–150 kg group had the highest rates of overweight-obesity (P < 0.001). University graduates had lower rates of chronic conditions compared to those with high school or lower education, with significant differences observed across conditions (P < 0.001). Employment status also influenced disease prevalence, with retired individuals showing the highest rates of diabetes and hypertension (P < 0.001). Lastly, participants with lower incomes (<5,000 SAR) had fewer reported chronic conditions compared to those with higher incomes, though significant differences were observed across income groups (P < 0.001) As seen in Table 3, the distribution of vaccination across different doses showed that Pfizer/BioNTech was the most common vaccine for both the 1st and 2nd doses, with 83.46% and 78.1% of participants receiving it, respectively (P < 0.001 for both). Oxford/AstraZeneca was the second most frequently administered vaccine, with 15.5% for the 1st dose and 13.19% for the 2nd dose. Moderna was used more for the 3rd dose (13.44%) compared to the earlier doses. Notably, 43.67% of participants received “Other Vaccines” for the 3rd dose, making it the most common for this stage. Janssen and other vaccine types had minimal to no uptake across all doses. Vaccine manufacturer (P < 0.001) observed significant differences across the distribution of doses.

**TABLE 3:** Distribution of vaccination by vaccine manufacturer for different doses.

S.No	Vaccine manufacturer	1st dose (N, %)	95% CI	Sig
1	Pfizer/BioNTech	323 (83.46%)	(0.7976–0.8716)	P < 0.001
2	Oxford/AstraZeneca	60 (15.5%)	(0.119–0.1911)	
3	Janssen (Johnson & Johnson)	0 (0%)	(0–0)	
4	Moderna	4 (1.03%)	(0.0003–0.0204)	
5	Other Vaccines*	0 (0%)	(0–0)	

S.No	Vaccine Manufacturer	2nd Dose (N, %)	95% CI	Sig
1	Pfizer/BioNTech	296 (78.1%)	(0.7398–0.8222)	P < 0.001
2	Oxford/AstraZeneca	50 (13.19%)	(0.0982–0.1656)	
3	Janssen (Johnson & Johnson)	3 (0.79%)	(0.0009–0.0167)	
4	Moderna	30 (7.92%)	(0.0523–0.1061)	
5	Other Vaccines*	0 (0%)	(0–0)	

S.No	Vaccine Manufacturer	3rd Dose (N, %)	95% CI	Sig
1	Pfizer/BioNTech	152 (39.28%)	(0.3436–0.4419)	P < 0.001
2	Oxford/AstraZeneca	6 (1.55%)	(0.0031–0.0279)	
3	Janssen (Johnson & Johnson)	0 (0%)	(0–0)	
4	Moderna	52 (13.44%)	(0.1–0.1687)	
5	Other Vaccines*	169 (43.67%)	(1.3868–0.4866)	

*Other Vaccines: Mixture of Pfizer-BioNTech, and Oxford-AstraZeneca.

The results presented in Table 4 indicate a notable association between vaccine manufacturers and post-vaccination side effects following both the first and second doses. After the first dose, 36.53% of participants who received the Pfizer/BioNTech vaccine reported side effects, with 43.03% requiring some medication, while only 5.57% of those who received the Oxford/AstraZeneca vaccine reported side effects. The duration of side effects also varied, with 53.87% of Pfizer/BioNTech recipients experiencing side effects lasting 1–2 days. In contrast, only 9.6% of Oxford/AstraZeneca recipients reported similar short-term side effects. The p-values for these associations were not statistically significant (P > 0.05), indicating that while there are observable trends, the differences may not be clinically meaningful.

**TABLE 4:** Associations between vaccine manufacturers and post-vaccination side effects.

Vaccines association with side effects after 1st dose						
S.No	Vaccine manufacturer	No. Side effects (N, %)	No medication needed (N, %)	Some medication needed (N, %)	Visited doctor or hospitalized (N, %)	P-value
1	Pfizer/BioNTech	118 (36.53%)	60 (18.58%)	139 (43.03%)	6 (1.86%)	0.192
2	Oxford/AstraZeneca/Others*	18 (5.57%)	9 (2.79%)	34 (10.53%)	3 (0.93%)	

Vaccines association with duration of Side Effects after 1st dose						
S.No	Vaccine Manufacturer	1–2 days (N, %)	3–6 days (N, %)	Week or more (N, %)	No Side effects (N, %)	P-Value
1	Pfizer/BioNTech	174 (53.87%)	76 (23.53%)	9 (2.79%)	64 (19.81%)	0.297
2	Oxford/AstraZeneca/Others*	31 (9.6%)	21 (6.5%)	3 (0.93%)	9 (2.79%)	

Vaccines association with Side Effects after 2nd dose						
S.No	Vaccine Manufacturer	No. Side Effects (N, %)	No medication needed (N, %)	Some medication needed (N, %)	Visited doctor or Hospitalized (N, %)	P-Value
1	Pfizer/BioNTech	114 (35.29%)	63 (19.5%)	104 (32.2%)	5 (1.55%)	0.131
2	Oxford/AstraZeneca/Jnassen	19 (5.88%)	10 (3.1%)	22 (6.81%)	1 (0.31%)	
3	Moderna/Others*	7 (2.17%)	3 (0.93%)	19 (5.88%)	0 (0%)	

Vaccines association with duration of Side Effects after 2nd dose						
S.No	Vaccine Manufacturer	1–2 days (N, %)	3–6 days (N, %)	Week or more (N, %)	No Side effects (N, %)	P-Value
1	Pfizer/BioNTech	161 (49.85%)	51 (15.79%)	8 (2.48%)	68 (21.05%)	0.153
2	Oxford/AstraZeneca/Jnassen	20 (6.19%)	17 (5.26%)	2 (0.62%)	13 (4.02%)	
3	Moderna/Others*	18 (5.57%)	6 (1.86%)	0 (0%)	5 (1.55%)	

*Other Vaccines: Mixture of Pfizer-BioNtech and Oxford-AstraZeneca.

Following the second dose, 35.29% of Pfizer/BioNTech recipients reported side effects, with 32.2% needing some medication. Comparatively, 5.88% of Oxford/AstraZeneca recipients experienced side effects, with a lower percentage requiring medication (6.81%). The duration of side effects after the second dose showed that 49.85% of Pfizer/BioNTech recipients reported side effects lasting 1–2 days, while only 6.19% of Oxford/AstraZeneca recipients reported similar durations. Again, the p-values indicated no statistically significant differences (P > 0.05). Overall, these findings suggest that while Pfizer/BioNTech is associated with a higher prevalence of reported side effects compared to other vaccines, the clinical significance of these differences remains uncertain.

The results in Table 5 show how COVID-19 infection rates changed following different vaccine doses. For Pfizer/BioNTech recipients, infections were highest before vaccination (43.18%), but dropped to 16.67% after the first dose, 31.82% after the second dose, and down to 8.33% after the third dose. Although this decline was not statistically significant (P = 0.928), it suggests a downward trend. In contrast, Oxford/AstraZeneca recipients had fewer infections overall, starting at 7.58% before vaccination and decreasing to 3.79% after the first dose, then stabilizing around 2.27% by the third dose.

**TABLE 5:** Associations between vaccine manufacturers and COVID-19 infections post-vaccination.

Vaccinces1st dose VS association with infection post vaccination						
S.No	Vaccine manufacturer	Before taking vaccine	After the 1st dose	After the 2nd dose	After the 3rd dose	P-value
1	Pfizer/BioNTech	57 (43.18%)	22 (16.67%)	42 (31.82%)	11 (8.33%)	0.928
2	Oxford/AstraZeneca/Others*	10 (7.58%)	5 (3.79%)	8 (6.06%)	3 (2.27%)	
Vaccinces2nd Dose VS associationwith infection post Vaccination						
1	Pfizer/BioNTech	49 (37.12%)	17 (12.88%)	35 (26.52%)	12 (9.09%)	0.34
2	Oxford/AstraZeneca/Others*	17 (12.88%)	9 (6.82%)	14 (10.61%)	1 (0.76%)	
Vaccinces3rd Dose VS associationwith infection post Vaccination						
1	Pfizer/BioNTech	24 (18.18%)	11 (8.33%)	8 (6.06%)	10 (7.58%)	0.000*
2	Oxford/AstraZeneca/Others*	42 (31.82%)	15 (11.36%)	41 (31.06%)	3 (2.27%)	

P < 0.05, Pearson Chi-square test, *Other Vaccines: Mixture of Pfizer-BioNtech and Oxford-AstraZeneca.

After the second dose, Pfizer/BioNTech recipients again showed a decrease in infection rates, from 37.12% pre-vaccination to 12.88% after the first dose, though the rate rose slightly to 26.52% after the second dose (P = 0.34). Oxford/AstraZeneca recipients experienced a similar trend, with infections dropping from 12.88% before vaccination to 6.82% after the first dose, before rising slightly to 10.61% after the second dose.

The third dose brought the most notable changes, with Pfizer/BioNTech recipients showing a clear reduction in infection rates—from 18.18% before vaccination to 8.33% after the first dose, 6.06% after the second dose, and a slight increase to 7.58% after the third dose, which was statistically significant (P = 0.000). Oxford/AstraZeneca recipients, starting with a higher initial infection rate (31.82%), saw a consistent decline, reaching 2.27% after the third dose. These results emphasize the effectiveness of the third dose, especially for Pfizer/BioNTech, in lowering COVID-19 infection rates.

The results presented in Table 6 provide a comparative analysis of the impact of COVID-19 vaccination on infection rates among participants. In the first section, individuals who reported not being vaccinated had a significantly higher rate of COVID-19 infections, with 85.71% indicating they had been infected compared to only 14.29% who had never been infected (P = 0.029). In contrast, among those who had been vaccinated, 56.33% reported having been infected, while 43.67% had never experienced an infection. This suggests that vaccination is associated with a reduced likelihood of experiencing COVID-19 infections.

**TABLE 6:** A comparative analysis of impact of COVID-19 vaccination on infection rates.

Vaccination status vs. never got infection with COVID-19				
	Vaccination status	Have you ever been infected with COVID-19?		
		No (N, %)	Yes (N, %)	P-value
Have you been vaccinated with any COVID-19 vaccine?	No	2 (14.29%)	12 (85.71%)	0.029*
	Yes	169 (43.67%)	218 (56.33%)	

Vaccination status vs. Infection with COVID-19 BEFORE taking vaccine				
	Vaccination status	Have you been infected before taking any COVID-19 Vaccine?		
		No (N, %)	Yes (N, %)	P-Value
Have you been vaccinated with any COVID-19 Vaccine?	No	12 (85.71%)	2 (14.29%)	0.604
	Yes	310 (80.1%)	77 (19.9%)	

Vaccination status vs. Infection with COVID-19 AFTER taking vaccine				
	Vaccination status	Have you been infected after taking any COVID-19 Vaccine?		
		No (N, %)	Yes (N, %)	P-Value
Have you been vaccinated with any COVID-19 Vaccine?	No	14 (100%)	0 (0%)	0.020*
	Yes	278 (71.83%)	109 (28.17%)	

*P < 0.05, Pearson Chi-square test.

In the second section, the analysis of infection rates before vaccination showed that a majority of both vaccinated (80.1%) and unvaccinated (85.71%) individuals had not been infected prior to receiving the vaccine, with no statistically significant difference (P = 0.604). However, the third section highlights a critical finding regarding post-vaccination infections: all unvaccinated individuals (100%) reported no infections after vaccination, while 28.17% of vaccinated individuals experienced infections post-vaccination (P = 0.020). This indicates that while vaccination does not completely eliminate the risk of infection, it is associated with a lower incidence of COVID-19 infections after vaccination compared to those who remain unvaccinated.

## Discussion

This study aimed to explore the impact of different COVID-19 vaccines on adverse effects, infection rates, and vaccine hesitancy in region of Hail Saudi Arabia, with a particular focus on sociodemographic factors such as age, gender, and existing health conditions. The findings shed light on various trends related to vaccine safety, effectiveness, and the role of vaccination in managing COVID-19, especially among those with chronic conditions.

The analysis revealed significant disparities in chronic disease prevalence among different groups. Women generally reported more chronic health issues than men, with higher rates of heart disease, diabetes, and hypertension (P < 0.001). In contrast, men exhibited a slightly higher prevalence of overweight and obesity. Saudi nationals experienced a higher burden of chronic diseases compared to non-Saudis, indicating that underlying health conditions may influence vaccine uptake and effectiveness (Riad et al., 2021; Al-Mohaithef et al., 2021; Huang et al., 2020; Fadini et al., 2020). Age and weight also played a crucial role; older participants and those with higher body weight had significantly higher rates of diabetes and hypertension. Furthermore, lower educational attainment and income were associated with increased diabetes rates, suggesting that socio-economic factors impact chronic disease management and access to healthcare services (Al-Mohaithef et al., 2021). The identification of these diseases within our research cohort emphasises the necessity for the implementation of focused vaccination programmes and preventative interventions for populations at elevated risk (Földi et al., 2020).

Regarding vaccine preferences, the Pfizer/BioNTech vaccine was the most widely administered among participants, reflecting a strong public trust in mRNA vaccines. Over 80% of participants chose Pfizer/BioNTech as their first dose, with similar uptake rates for subsequent doses (P < 0.001). This mirrors global patterns, where mRNA vaccines are often favored due to perceived efficacy and availability (Baden et al., 2021; Mattiuzzi and Lippi, 2022; Schwarzinger et al., 2021). On the other hand, Oxford/AstraZeneca was less preferred, accounting for 15.5% and 13.19% of the first and second doses, respectively. The lower uptake of Janssen and Moderna vaccines may be attributed to factors like limited availability and differing public perceptions of their effectiveness (Schwarzinger et al., 2021).

The study also observed significant differences in post-vaccination side effects between vaccine types. Pfizer/BioNTech recipients reported a higher incidence of side effects compared to those receiving Oxford/AstraZeneca, particularly after the first dose, where 36.53% experienced side effects, compared to 5.57% of Oxford/AstraZeneca recipients. The duration of side effects also varied, with most Pfizer/BioNTech recipients reporting symptoms lasting 1–2 days, while the majority of Oxford/AstraZeneca recipients experienced shorter side effects (Riad et al., 2021; Chapin-Bardales et al., 2021). However, when analyzed across all doses, the differences in side effect prevalence among vaccine manufacturers were not statistically significant. These findings align with existing research, reinforcing the general safety and tolerability profile of COVID-19 vaccines (Polack et al., 2020). Nonetheless, these results highlight the reactogenicity of mRNA vaccines, reinforcing the need for healthcare providers to inform individuals about potential side effects to mitigate vaccine hesitancy (Alzamil et al., 2023).

The effectiveness of vaccination in reducing infection rates post vaccination was evident, with a significant drop in infections among Pfizer/BioNTech recipients from 43.18% before vaccination to 8.33% after the third dose. However, the lack of statistical significance (P = 0.928) suggests that while there is a trend toward reduced infections, the differences may not be clinically meaningful. Yet, it could indicate that other factors, such as individual immune responses and exposure risks, may also influence infection rates (Al-Mohaithef et al., 2021; Jamous et al., 2023). This aligns with findings from other studies that emphasize the importance of ongoing evaluations of vaccine efficacy, particularly as new variants emerge (Moussa et al., 2023). Elamin et al. examined the effectiveness of two vaccines—Pfizer/BioNTech and AstraZeneca—in Saudi Arabian adults from the Jazan Region. The findings demonstrated that after 6 months, the Pfizer/BioNTech and ASZ vaccines’ ability to prevent infection was increased by up to 30%. (Elamin et al., 2023). Contrast to other studies Mohammed et al. reported higher percentage of COVID−19 infection post vaccination after taking shots of Pfizer/BioNTech and Oxford/AstraZeneca vaccine (Mohammed et al., 2023). After performing a study in Saudi Arabia, Alsaiari et al. concluded that individuals should be warned that they are likely to encounter at least a single adverse effect. The most common side effects reported were headaches, fevers, musculoskeletal pain, and transient post-injection inflammation. However, most people have mild to moderate manifestations, and the side effects disappear quickly (Alsaiari et al., 2023). Similarly, Alhazmi et al. and Zarei et al., noted that while Pfizer/BioNTech is effective in reducing infections, it is also linked to a higher rate of side effects (Alhazmi et al., 2021; Zarei et al., 2022). Additionally, the study’s results regarding vaccine hesitancy highlight the complex interplay between sociodemographic factors and public health outcomes. Factors such as age, gender, education, and income significantly influenced health outcomes and vaccine acceptance, reinforcing the need for targeted public health strategies that consider these variables (Albeladi et al., 2021).

The analysis further indicated that vaccinated individuals had significantly lower infection rates compared to unvaccinated participants, with only 56.33% of vaccinated individuals reporting infections, versus 85.71% of unvaccinated individuals (P < 0.029). This emphasizes the effectiveness of vaccination in reducing the risk of COVID-19 infection. However, infections were not completely eliminated among vaccinated individuals, with 28.17% reporting infections after vaccination. This finding aligns with studies that show booster doses are essential in maintaining immunity and protecting against emerging variants (Alobaidi, 2021; Qattan et al., 2021).

The study’s strengths include a large and diverse sample size, capturing a broad spectrum of the Saudi population and providing a comprehensive understanding of vaccine-related outcomes. The study’s focus on sociodemographic factors also allowed for a nuanced analysis of how different characteristics affects vaccine safety and effectiveness. However, there are several limitations to consider. First, our reliance on self-reported data may introduce recall bias and social desirability bias, potentially affecting the accuracy of reported side effects and infection history (Dalziel et al., 2018). In addition, although we used cluster random sampling, participants were recruited from specific locations within the Hail region, which may result in selection bias and limit the generalizability of our findings to the entire Saudi population. Moreover, the cross-sectional design of the study captures only a snapshot of vaccine effects at one point in time, limiting our ability to infer cause-and-effect relationships or assess long-term outcomes. Future research should consider longitudinal studies incorporating clinical data to provide a more comprehensive analysis of vaccination impacts and long-term effects.

## Conclusion

This study offers valuable insights into COVID-19 vaccination outcomes in Saudi Arabia, confirming that vaccines, particularly mRNA types like Pfizer/BioNTech, play a crucial role in reducing infection rates and preventing severe outcomes. Despite a higher rate of side effects associated with mRNA vaccines, their effectiveness in lowering infection rates is evident. These findings support the importance of ongoing public health campaigns that emphasize clear communication about vaccine safety and the benefits of vaccination, particularly in terms of booster doses and protection against new variants (Al-Mohaithef et al., 2021; Prasad et al., 2021). Addressing vaccine hesitancy through transparent information is critical for improving vaccine acceptance, especially in populations with higher levels of hesitancy or lower access to healthcare, these efforts can enhance public trust, increase vaccine coverage, and improve overall public health outcomes in Saudi Arabia.

## Data Availability

The original contributions presented in the study are included in the article/supplementary material, further inquiries can be directed to the corresponding author.
